# Arachidonic Acid Enhances Turnover of the Dermal Skeleton: Studies on Zebrafish Scales

**DOI:** 10.1371/journal.pone.0089347

**Published:** 2014-02-19

**Authors:** Erik de Vrieze, Mari Moren, Juriaan R. Metz, Gert Flik, Kai Kristoffer Lie

**Affiliations:** 1 Department of Organismal Animal Physiology, Radboud University Nijmegen, Nijmegen, The Netherlands; 2 Institute for Water and Wetland Research, Radboud University Nijmegen, Nijmegen, The Netherlands; 3 NIFES (National Institute of Nutrition and Seafood Research), Bergen, Norway; National University of Singapore, Singapore

## Abstract

In fish nutrition, the ratio between omega-3 and omega-6 poly-unsaturated fatty acids influences skeletal development. Supplementation of fish oils with vegetable oils increases the content of omega-6 fatty acids, such as arachidonic acid in the diet. Arachidonic acid is metabolized by cyclooxygenases to prostaglandin E2, an eicosanoid with effects on bone formation and remodeling. To elucidate effects of poly-unsaturated fatty acids on developing and existing skeletal tissues, zebrafish (*Danio rerio*) were fed (micro-) diets low and high in arachidonic acid content. Elasmoid scales, dermal skeletal plates, are ideal to study skeletal metabolism in zebrafish and were exploited in the present study. The fatty acid profile resulting from a high arachidonic acid diet induced mild but significant increase in matrix resorption in ontogenetic scales of adult zebrafish. Arachidonic acid affected scale regeneration (following removal of ontogenetic scales): mineral deposition was altered and both gene expression and enzymatic matrix metalloproteinase activity changed towards enhanced osteoclastic activity. Arachidonic acid also clearly stimulates matrix metalloproteinase activity *in vitro*, which implies that resorptive effects of arachidonic acid are mediated by matrix metalloproteinases. The gene expression profile further suggests that arachidonic acid increases maturation rate of the regenerating scale; in other words, enhances turnover. The zebrafish scale is an excellent model to study how and which fatty acids affect skeletal formation.

## Introduction

Suboptimal nutrition in fish aquaculture may cause skeletal deformities that have major economic and ethical implications (reviewed by Boglino *et al.* [1]). Essential fatty acids, in particular the long chained poly-unsaturated fatty acids (PUFAs), influence fish larval development. Effects can be mediated both via the level of PUFAs and via the ratio between omega-3 and omega-6 fatty acids [1], [2]. High levels of arachidonic acid (ARA, 20:4(ω-6)) have been linked to malpigmentation [3], [4] and skeletal deformities [1], [5]. Changes in dietary PUFA content can affect bone health of teleost fish (and mammals) and is reflected in bone cell make-up and matrix quality [6]–[10]. Dietary PUFA deficiency impairs synthesis of bone matrix and facilitates bone demineralization and resorption [11], [12]. Surprisingly, changes in the dietary fatty acid profile can also have detrimental effects on both mammals [13], [14] and fish [5], [7]. High dose dietary fatty acid supplementation results in lower bone mineral density, decreased rate of bone formation and skeletal malformations. In aquaculture, this becomes particularly apparent as deformities during the early skeletal development [15], urging the need to pursuit optimal dietary PUFA content.

Fish oils in aquaculture feeds are increasingly replaced by vegetable oils to reduce cost and pressure on wild fish stocks [16]. The high omega-6 PUFA content in vegetable oils can however have dramatic effects on the teleost skeleton as desaturase and elongase enzymes metabolize omega-6 PUFAs into ARA [6], [8], [10]. Cyclooxygenases convert ARA into prostaglandin E2 (PGE2), an eicosanoid with potent regulatory actions in bone physiology and stress- and immune responses [17], [18]. In contrast, omega-3 fatty acids, enriched in fish oils (*e.g.* eicosapentaenoic acid; EPA, 20:5(ω-3)), are converted into the less potent eicosanoid prostaglandin E3 (PGE3) [13]. An increase in ARA may therefore alter signaling pathways in bone cells via increased PGE2 production. During mechanical loading of mammalian bone, osteocytes and mature osteoblasts release PGE2 as a signal to stimulate bone resorption [19], [20]. PGE2 shifts the balance between the production of osteoclast-stimulating, osteoblast-derived RANKL (receptor activator of nuclear factor kappa-B ligand) and its decoy receptor OPG (osteoprotegerin) towards increased RANKL-signaling and osteoclast activation [17], [21]. ARA further attenuates alkaline phosphatase (ALP; an osteoblast marker of bone formation) activity *in vitro*, while it stimulates secretion and expression of matrix metalloproteinase 9 (MMP-9; an osteoclast-derived collagen-degrading enzyme). Both effects, described for cultured mammalian cells, are believed to be indirect and mediated by PGE2 [22], [23]. Moreover, mineralization of the extracellular matrix by VSa16 cells (osteoblast-like cells from gilthead seabream vertebra) has been shown to decrease dramatically upon exposure to ARA, corroborated by a decreased expression of several bone matrix genes [24]. In mammalian bone, PGE2 stimulates osteoblastogenesis and osteoclast activity simultaneously, thus increasing the rate of bone remodeling [21], [25]. Likewise, goldfish scales treated with prostaglandin E2 responded with increased enzyme activity of typical osteoblast and osteoclast associated phosphatases [26]. Clearly, both osteoblasts and osteoclasts are targets of ARA and/or PGE2.

The zebrafish is a valued model in biomedical bone research [27], [28], but as yet surprisingly underappreciated as a model for other teleost fish species. All the well-known advantages of zebrafish over numerous mammalian models apply evenly well to aquaculture research. So far few studies, if any, looked at the zebrafish requirement of essential nutrients. We here report how essential fatty acids affect growth and metabolism of the zebrafish scale, which is a superb model to study the dynamics of mineralized skeletal tissues in adult fish [29], [30]. Zebrafish, like many other teleost fish, possess scales of the elasmoid type. Elasmoid scales provide the researcher with a sample of mineralized collagen matrix and associated scleroblasts and osteoclasts [29]. The scleroblast (scale-forming cell) is often referred to as osteoblast, since the similarity with mammalian osteoblasts, *e.g.* in their respective transcriptomes, becomes increasingly apparent (reviewed by Metz et al. [29]). Although scales are considered dentin-derived tissues, and thus not regarded bone [31], their abundance on a single fish and ease of handling does make them an attractive alternative model to study the dynamics of skeletal formation. Over the last decade, it has been shown that scale cells, treated with hormones such as parathyroid hormone (PTH), calcitonin, prolactin, glucocorticoids and estrogens, responded as anticipated from mammalian bone studies [32]–[36]. This indicates that processes and signaling pathways involved in matrix deposition, mineralization and matrix resorption are shared between bone cells and elasmoid scale cells.

As teleost fish continue to grow throughout their life, and the scale compartment is an important calcium and phosphorus reservoir [15], [37], it may come as no surprise that demineralization of the scale matrix occurs [38]. Elasmoid scales are acellular, *i.e.* cells are not entrapped within, but lined along the collagenous scale matrix. When a scale is removed, lining cells that remain in the scale pocket differentiate to start formation of a new scale [29], [30], [39]. In zebrafish kept at 28°C, deposition of new scale matrix starts within two days and within a matter of weeks, the new scale is fully regrown and mineralized [36], [38]. The regeneration paradigm thus offers a model to study principles underlying early skeletal development in an adult fish.

We here present the zebrafish scale as a practical model system to study effects of dietary fatty acid supplementation on the skeleton. Adult fish were fed a diet either low or high in ARA for four weeks. Scale mineralization and markers of matrix formation and resorption were investigated to elucidate the effects of ARA on the existing skeleton. In addition, regenerating scales were used to investigate the effects of ARA on skeletal growth and development.

## Materials and Methods

### Ethics statement

This study was carried out in strict accordance with directive of the European Union on the protection of animals used for scientific purposes. The protocols were approved by the Committee on the Ethics of Animal Experiments of the Radboud University (Permit Numbers: RU-DEC2010-105; RU-DEC2011-266). Removal of scales was always done under 2-phenoxyethanol anesthesia, and all efforts were made to minimize suffering.

### Formulation and preparation of diets

Two mixes of different oils were used to prepare diets containing low and high levels of ARA, respectively. The oils were combined so that each of the two diets contains equal amounts of DHA (docosahexaenoic acid, 22:6(ω-3)), but different levels of ARA. Diets were prepared using the ingredients listed in table 1 and as described previously by Halver [40]. Compositions of mineral, vitamin and amino acid mixes used to prepare the diets are listed in tables S1, S2 and S3 in File S1, respectively.

**Table 1:** Zebrafish feed composition.

Ingredients	Low ARA diet (g/kg)	High ARA diet (g/kg)
Casein^1^	480	480
Gelatin^2^	119	119
Dextrin^3^	125	125
Cellulose^4^	50	50
Fish oil^5^	55	33
ARA-rich oil (Vevodar oil)^6^	-	77
Canola oil^7^	55	-
Mineral mix*	40	40
Vitamin mix*	10	10
Amino acid/betaine mix*	50	50
Astaxanthin^8^	1	1
Lecithin^4^	15	15

1 MP Biomedical, Solon, OH, USA, cat. no. 02901293;

2 Idun Industri AS, Skjetten, Norway;

3 Fluka Chemie, Buchs, Switzerland cat. no. 31400;

4 Sigma-Aldrich, St Louis, MO, USA;

5 Møllers tran, Oslo, Norway;

6 DSM nutritional products, Oslo, Norway;

7 Eldorado, Oslo, Norway;

8 Carophyll Pink, Hoffman-La Roche, Basle, Switzerland;

*Details on mineral mix, vitamin mix and amino acid mix composition can be found in supplementary tables 1, 2 and 3.

### Experimental setup

Adult zebrafish (*Danio rerio*, tupfel long fin) of 10 months old, with an average weight of 0.44±0.084 gram, were kept in 2-L tanks of nine fish each in a recirculating system. Four tanks were fed the ARA-restricted diet (low ARA), and four tanks were fed the ARA-enriched diet (high ARA). Fish were left undisturbed and fed twice daily at a total ration of 0.12±0.02 grams (2% of the estimated bodyweight) for four weeks. After 4 weeks of applying one of the two diets, from all fish approximately 50 scales were removed from the left flank under mild anesthesia (0.05% (v/v) 2-phenoxy-ethanol, Sigma Aldrich, Carlsbad, USA) to induce scale regeneration (day 0).

After 4, 7, 10 and 14 days of regeneration, all nine fish from one tank provided with low ARA diet and one with high ARA diet were sacrificed. Regenerating scales were sampled from all groups and stored for different analyses. Anaesthetized whole fish were frozen at −80°C for whole-body fatty acid analyses.

### Fatty acid analysis

Whole body and dietary fatty acid compositions were analyzed using a gas liquid chromatography (GLC) method previously described by Lie and Lamberdsen [41].

### Calcium and phosphorus analysis

Per fish, ten scales (with cells attached), either ontogenetic or regenerating, of the same individual fish were pooled, dried and dissolved in 200 µl nitric acid (65%). The thus obtained sample was diluted 100× with ultrapure water. Calcium, phosphorus and magnesium content were measured in duplicate with ICP-MS (inductively coupled plasma mass spectrometry; Thermo Fisher Scientific, Waltham, USA). Four measurements (on a total of 220) with duplicates that varied more than 15% were discarded.

### Histochemistry and morphometric analysis

Regenerating and ontogenetic scales were fixed overnight in 4% paraformaldehyde in PBS (phosphate-buffered saline) at 4°C and, following two wash steps with PBS, stained for either mineralization or TRAcP (tartrate-resistant acid phosphatase). To stain calcium phosphates, according to Von Kossa, fixed samples were rinsed with MilliQ water and subsequently incubated for 1 hour in 5% (w/v) silver nitrate under strong light. Scales were then washed twice with MilliQ water and fixed for 5 minutes in 5% (w/v) sodium thiosulfate, rinsed again, mounted and coverslipped. TRAcP activity staining was done as recently described in detail [36].

All samples were photographed under a Leica DMRBE microscope with a PFC-450C camera (Leica microsystems, Wetzlar, Germany) and morphometric analysis was done with Photoshop (Adobe, San Jose, USA). The scale surface area was measured as the total area within the perimeter of a Von Kossa-stained scale. Measurement of the resorption pits included all regions within the perimeter of the scale that were not stained for mineralization with the exception of the unmineralized grooves (*radii*) that radiate from the centre focus. Quantification of the TRAcP activity staining included both the surface covered by osteoclast as well as the TRAcP secreted in the scale matrix.

### Scale culture and gelatin zymography

Scales obtained from the feeding trial were cultured in minimal essential culture medium (MEMα) supplemented with 1% (v/v) penicillin/streptomycin (Invitrogen) for 22 hours at 28°C. The entire supernatant was then reduced to a volume 10 µl by cryo-sublimation under vacuum. For zymography, the entire samples were loaded onto SDS-PAGE gels supplemented with 1 mg/ml gelatin as described by de Vrieze et al [38]. After staining with Coomassie blue, the gels were preserved between two cellophane sheets, scanned and band densities were analyzed with Photoshop (Adobe).

### In-vitro scale cultures with arachidonic acid

Arachidonic acid (cat.no A355; Sigma-Aldrich) was coupled to fatty acid-free albumin (PAA, Pasching, Austria) by addition of 0.04 ml chloroform per mg ARA as described by Lie and Lambertsen [41]. The chloroform was then evaporated under a flow of nitrogen gas after which the ARA stock was stored in −80°C until use. Ontogenetic scales were picked from eight adult zebrafish. The scales were washed six times in 0.4 ml L-15 medium supplemented with 1% (v/v) penicillin/streptomycin prior to incubation in treatment media. Pools of 10 scales (obtained from one fish) were incubated in 0.3 ml L-15 with 1% penicillin/streptomycin. Pooled scales from each individual fish were cultured in 48-wells plates at four different concentrations of (albumin-coupled) arachidonic acid: 0 (control), 5, 50 and 150 µM ARA. The concentration of albumin was adjusted when necessary, to equal total protein in all treatment groups. The scales were incubated at 28°C under gentle agitation for 24 or 48 hours. Ten microliters of the culture medium from each well was used for Mmp2 and Mmp9 gelatin zymography after 24 h and 48 h of incubation.

### Gene expression analysis

Scales (including their cells lining the scale surface) were homogenized in Qiazol (Qiagen, Hilden, Germany) and total RNA was extracted using BioRobot EZ1 and RNA Tissue Mini Kit (Qiagen) according to the manufacturer's instruction. DNase treatment was included in the protocol to avoid DNA contamination. The quantity and purity of total RNA were assessed by NanoDrop ND-1000 spectrophotometry (NanoDrop Technologies, Wilmington, Germany). All samples had 260/230 ratios over 1.6 and 260/280 ratios over 1.8 implying a high purity of RNA. The RNA integrity (RIN) was measured with RNA 6000 Nano LabChip kits and the Agilent 2100 Bioanalyzer (Agilent Technologies, Palo Alto, USA) and was found to be over 8.0. Copy DNA was synthesized in duplicate in a 20-µl reaction volume for each biological replicate using the Superscript II Reverse transcriptase kit (Invitrogen) and 250 ng total RNA as input for each reaction. For downstream qPCR efficiency calculations, two-fold serial dilutions (500 ng – 15.6 ng) using pooled total RNA from all scales were prepared and reverse transcribed on each 96-wells plate. No amplification control (NAC) and no template control (NTC) were added to each plate to demonstrate absence of genomic contamination in the samples and absence of template contamination in the reagents. Reverse transcription was performed at 25°C for 10 min, followed by 42°C for 1 h in a GeneAmp PCR 9700 machine (Applied Biosystems, Foster City, USA). Quantitative PCR analysis and crossing point calculations were performed as previously described by Lie and Moren [42]; the primers used are listed in table S4 in File S1. Mean normalized expression (MNE), using *rpl13a* and *tuba1* as reference genes, was calculated individually for ontogenetic and regenerating scales, using GeNorm software [43].

## Results

### Whole body fatty acid profiles

At the end of the entire experiment, after feeding the experimental diets for six weeks, the fatty acid profile of the zebrafish had changed predictably and according to our intention between the groups (Table 2). There was a twenty-fold difference in ARA content between the low and high ARA fed groups. The EPA/ARA ratios were 3.8 and 0.4, respectively, in the low and high ARA diet fed groups and there was very little difference in whole body DHA content between the two diets as intended. The feeding regime did not result in differences in growth of the fish; there were no significant differences in weight at the end of the experiment (data not shown).

**Table 2:** Fatty acid profiles of the diets and of the zebrafish fed the respective diets for 6 weeks.

	Diet		Fish		
	Low ARA	High ARA	Low ARA	High ARA	P-value
Sum unidentified %	1.6	1.2	1.29±0.05	1.16±0.02	0.4562
Sum identified %	98.4	98.8	98.71±0.05	98.84±0.02	0.4562
Sum saturated %	13.2	20.6	20.33±0.25	24.04±0.12	<0.001
Sum mono-unsaturated %	53.2	30.8	46.54±0.46	33.84±0.20	<0.001
Sum poly-unsaturated%	32	47.4	31.87±0.24	40.96±0.18	<0.001
18:3ω-3%	5.3	0.6	4.53±0.07	2.67±0.04	<0.001
20:5ω-3 (EPA) %	4.6	2.5	2.59±0.06	1.31±0.01	<0.001
22:6ω-3 (DHA) %	6	3.3	8.06±0.12	6.01±0.10	<0.001
Sum ω-3%	18.5	7.6	18.40±0.24	12.24±0.12	<0.001
20:3ω-6%	<0.1	2.1	0.37±0.04	1.68±0.02	<0.001
20:4ω-6 (ARA) %	0.3	27	0.67±0.41	15.04±0.17	<0.001
Sum ω-6%	13.3	39.8	13.38±0.44	28.66±0.17	<0.001
Sum EPA+DHA %	10.6	5.7	10.63±0.16	7.31±0.09	<0.001
(ω-3)/(ω-6)	1.4	0.2	1.38±0.13	0.43±0.05	<0.001

Results of the zebrafish fatty acid profiles are expressed as average ± SEM of 9 fish. Statistical analysis was done with the Student's *t*-test.

### Ontogenetic scale physiology

**Figure 1:**
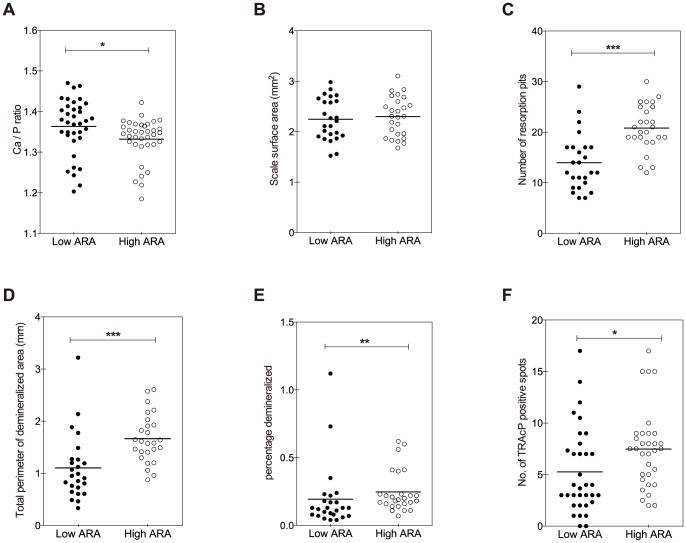
Effects of the different diets on scale mineralization. Analyses of the mineralized layer of ontogenetic scales were done after feeding the low ARA diet (solid dots) or the high ARA diet (open dots) for 4 weeks. ***A***: Calcium:phosphorus molar ratios were significantly lower in high ARA fed fish. ***B***: Surface area of the scales was unaffected. ***C***: The number of resorption pits per individual scale was higher in the high ARA fed fish. ***D***: Indeed, total perimeter of resorption pits also increased. ***E***: Percentage of each scale that is demineralized (as a result of osteoclast activity) was also increased in high ARA fed fish. ***F***: Number of TRAcP-stained regions per scale. Each replicate is shown; the horizontal lines represent means of 36 replicates. Statistical analysis was done with the Students T-test (*** p<0.001) except for (***E***) which required non-parametric testing (Mann Witney-U test, ** p<0.01).

Scales sampled from fish fed the high ARA diet for four weeks had a significantly lower molar Ca:P ratio compared to scales from fish fed the low ARA diet (figure 1A). No differences were observed in size between scales sampled from either group (figure 1B). The percentage of resorbed scale area, characterized by absence of mineral staining, was increased in the high ARA group (figure 1C). Accordingly, the number and perimeter of resorption pits were significantly higher in scales from fish fed the high ARA diet (figure 1D, E). Furthermore, these scales had a significantly more areas of TRAcP activity staining, indicative of increased osteoclast activity (figure 1F).

**Figure 2:**
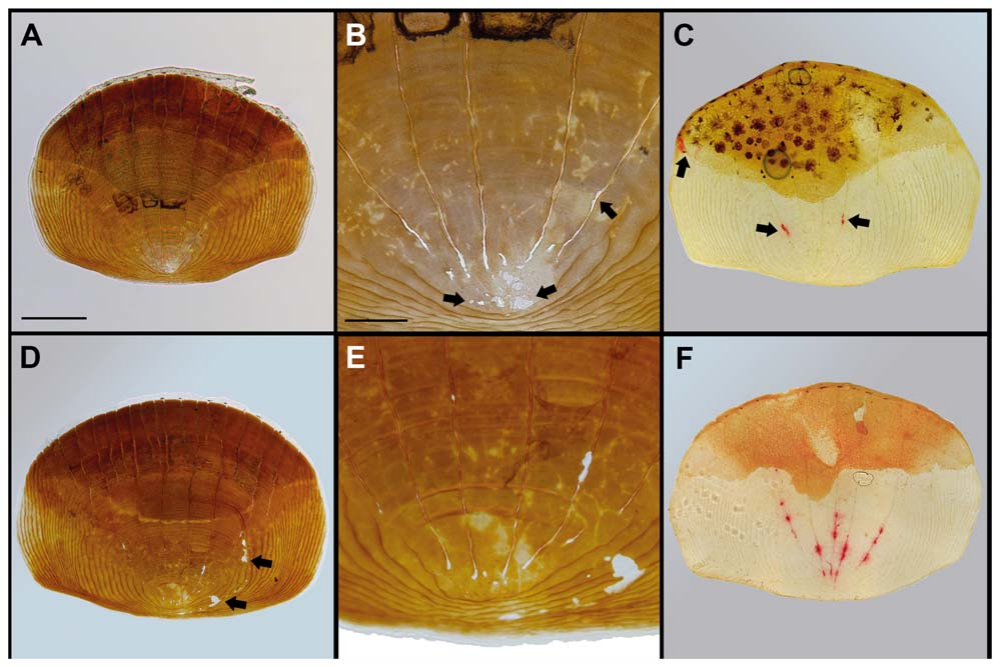
Effects of the different diets on scale mineralization. Representative examples of scales on which the quantifications from Figure 1 were based. ***A***: Example of a scale from the low ARA group stained for mineralization (Von Kossa staining, calcium phosphates stained brown). The scale is almost completely mineralized and the brown staining is only absent in the focus (lower central region) of the scale. Bar = 250 µm. ***B***: Detailed image of the focus of the scale shown in (***A***) with several unmineralized small resorption pits where staining is absent (arrows). Bar = 100 µm. ***C***: TRAcP activity-stained ontogenetic scale from the low ARA group with few red-stained areas positive for TRAcP activity, indicated by arrows. Bar = 250 µm. ***D***: Von Kossa stained ontogenetic scale from the high ARA group. Arrows indicate resorption pits outside the focus, seen more often in scales from high than from low ARA fed fish. Bar = 250 µm. ***E***: Detailed image of the scale shown in (***D***) with more resorption pits with typical round edges as a result of osteoclastic matrix degradation. Bar = 100 µm. ***F***: TRAcP activity-stained ontogenetic scale from the high ARA group with more spots stained red for TRAcP activity than in the low ARA group, indicating the presence of more osteoclasts in this group. Bar = 250 µm.

Representative pictures of the scales used for quantifications are shown in figure 2. Von Kossa stainings revealed some resorption pits, the obvious result of osteoclastic matrix degradation, on scales from fish fed the low ARA diet. Resorption pits were mainly restricted to the scale focus (figure 2A, B). Significantly more resorption pits were observed on scales from fish fed the high ARA diet and these were found more often in the periphery of the scale (figure 2D, E). TRAcP staining could be detected both in osteoclasts and in matrix, as a remnant of osteoclastic matrix resorption activity (figure 2C, F). Moderate TRAcP staining was found on scales from fish fed the low ARA diet (figure 2E). More TRAcP positive regions were observed in scales obtained from fish fed the high arachidonic acid diet (figure 2F).

**Figure 3:**
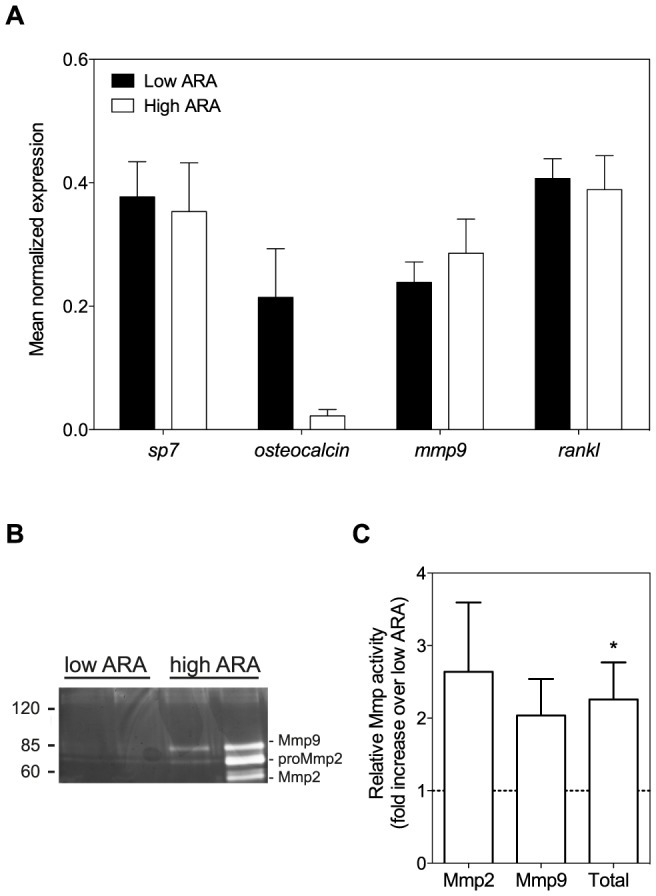
Gene expression profile and Mmp activity of ontogenetic scales after feeding the different diets. ***A*** *:* Gene expression analysis of ontogenetic scales from fish fed the low ARA diet (black bars) or the high ARA diet (white bars). No statistically different effects of the different diets were observed, not even in *osteocalcin* expression (P = 0.900; non-parametric test). Transcript abundance is expressed relative to an index of the reference genes *rpl13a* and *tuba1*. Results are displayed as means ± SEM of 16 scales randomly obtained from all of the 4 replicate tanks. ***B***: Example of a Coomassie-stained gelatin gel showing increased MMP activity in ontogenetic scales from the high ARA group (two lanes to the right) compared to the low ARA group (left lanes). Protein mass ladder is given in kiloDalton (kDa). ***C***: Semiquantitative analysis of gelatinolytic activity demonstrates that total of Mmp2 and Mmp9 activity, secreted by cultured ontogenetic scales, is higher in scales from fish fed the high ARA diet compared to the low ARA diet. The total amount of secreted gelatinolytic activity is significantly increased in the high ARA group (P<0.05; One sample T test). Results of high ARA samples are expressed relative to low ARA samples analyzed on the same gel. Bars represent the mean ± SEM of 8 samples.

The expression of several genes key in bone formation and resorption pathways were analyzed in ontogenetic scales from fish of both groups (figure 3A). No significant differences in gene expression could be detected between the two groups. For *osteocalcin* (also known as bone gla protein, *bgp*), the variation in expression within the low ARA group was particularly high.

Zymographic analysis of enzymatic Mmp2 and Mmp9 activities revealed increased band intensity in scale samples from the high ARA group compared to the low ARA group (figure 3B). This was confirmed by semi-quantitative band-intensity analysis, which further revealed that total gelatinolytic activity was significantly increased in scales of fish fed the high ARA diet (figure 3C).

### Regenerating scale physiology

**Figure 4:**
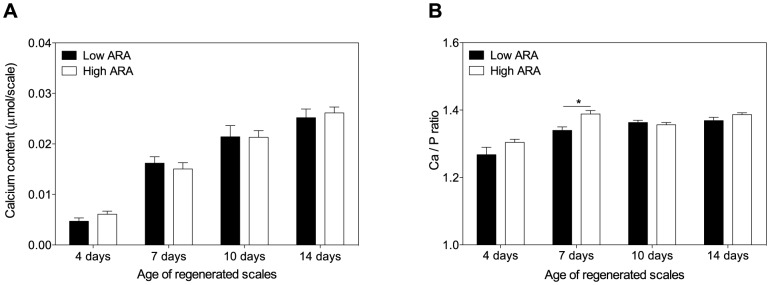
Analysis of scale mineralization during regeneration. ***A***: Absolute calcium contents in regenerating scales sampled at different time points after scale removal. Calcium content increases similarly in both groups as the scale grows during regeneration. ***B***: Calcium:phosphorus molar ratio of regenerating scales of different ages. The ratio increases in both groups from day 4 to 7, indicative of a changed mineral composition on the scale matrix. Effect of the diet is only observed on day 7; the Ca:P ratio in the high ARA groups is significantly increased compared to the low ARA group. Samples are expressed as mean ± SEM (N = 9); black bars represent samples obtained from fish fed the low ARA diet, and white bars represent samples from fish fed the high ARA diet. Statistical testing was conducted with the Students T-test (* p<0.05).

During regeneration, the contents of calcium, phosphorus and magnesium in the scale steadily increased, indicative of ongoing mineralization (shown for calcium; figure 4A). There was however no detectable difference in scale mineral content between low and high ARA fed groups. During scale regeneration, the Ca:P molar ratios were found increased in both groups between day 4 and day 7. After 7 days of regeneration, the Ca:P molar ratio was however significantly higher in scales from the high ARA group (figure 4B). Morphometric analysis of regenerating scales did not reveal any significant differences in size, perimeter or number of circuli between the two groups (data not shown).

**Figure 5:**
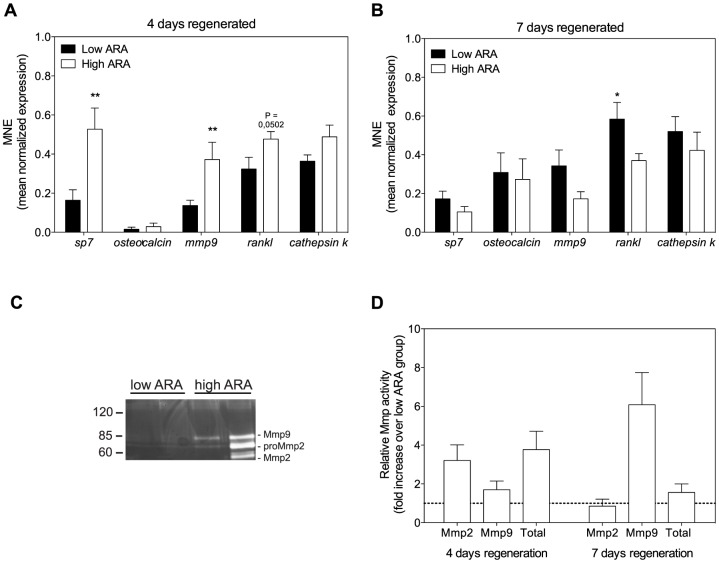
Gene expression profile and Mmp activity of regenerating scales of fish fed the different diets. ***A***: Gene expression of *sp7* and *rankl* (osteoblast), as well as *mmp9* (osteoclast) is higher in the high ARA group compared to the low ARA group in 4-day-old regenerating scales. ***B***: After 7 days of scale regeneration, these differences mostly disappeared. Gene expression of the low ARA group has increased compared to expression levels at day 4 and expression of *rankl* is also significantly higher compared to the high ARA group. Gene expression is shown as mean normalized expression (MNE). MNE for scales obtained from fish fed the ARA-restricted diet (low ARA) are shown with black bars and for scales from fish fed the ARA-enriched diet (high ARA) with white bars. Bars represent the mean ± SEM of 9 replicates. After assessment of normal distribution (D'Agostino & Pearson normality test), statistical analysis was done with either Student's *t*-test, or Mann-Whitney U-test (* p<0.05, ** p<0.01). ***C***: Enzymatic Mmp9 activity of 7-day-old regenerating scales is higher in the high ARA group, as seen on gelatin zymography. Protein mass ladder is depicted in kiloDalton (kDa). ***D***: Semiquantitative analysis of enzymatic Mmp2 and Mmp9 activity and total Mmp activity in 4-day-old and 7-day-old regenerating scales shows the increased Mmp9 activity observed in (***C***). Mmp activity of scales from high ARA fish is expressed relative to those of low ARA fish analyzed on the same gel. Bars represent the mean of 4 samples; horizontal line represents the low ARA samples. One sample *t*-test was used to compare the mean Mmp activity to 1. P-values for 4 days regeneration: P = 0.07 for Mmp2, P = 0.22 for Mmp9 and P = 0.06 for total. P-values for 7 days regeneration: P = 0.72 for Mmp2, P = 0.05 for Mmp9 and P = 0.29 for total.

In 4-days regenerated scales, significant changes were found in the gene expression profiles of osteoblast and osteoclast marker genes (figure 5A). Expression of the osteoblast-specific gene *sp7* (*osterix*), a transcription factor that activates a variety of genes involved in matrix formation and mineralization, was higher in scales from the high ARA group. Accordingly, *rankl*, the osteoblast-derived stimulator of osteoclastogenesis was upregulated in the high ARA group. Expression of *mmp9* had also increased in fish fed the high ARA diet. After 7 days of regeneration, the effects on *mmp9* expression were however reversed (figure 5B). After 7 days regeneration, expression of most genes was attenuated in the high ARA group compared to 4 days regeneration, with significant decreases in *sp7* and *rankl* expression (P<0.001 and P<0.05, respectively). In the low ARA group, expression of most genes was increased at day 7 of scale regeneration compared to day 4, with significant differences found for *osteocalcin*, *mmp9* and *rankl* (P<0.05). At 7 days regeneration, the only statistically significant difference in expression found between the two diets was in the expression of *rankl*, which was now lower in the high ARA group.

Gelatin zymography is indicative of the actual proteolytic activity of Mmp2 and Mmp9 (figure 5C), which are secreted by osteoclasts. On day 4, both total activity of secreted Mmps (P = 0.06), as well as individual Mmp2 (P = 0.07) and Mmp9 (P = 0.22) activity, was increased near statistical significance in scales sampled from fish fed the high ARA diet compared to fish fed the low ARA diet; on day 7 total gelatinolytic activity was increased in the high ARA group, which could completely be ascribed to Mmp9 (P = 0.05) (figure 5D).

### In-vitro exposure to ARA

**Figure 6:**
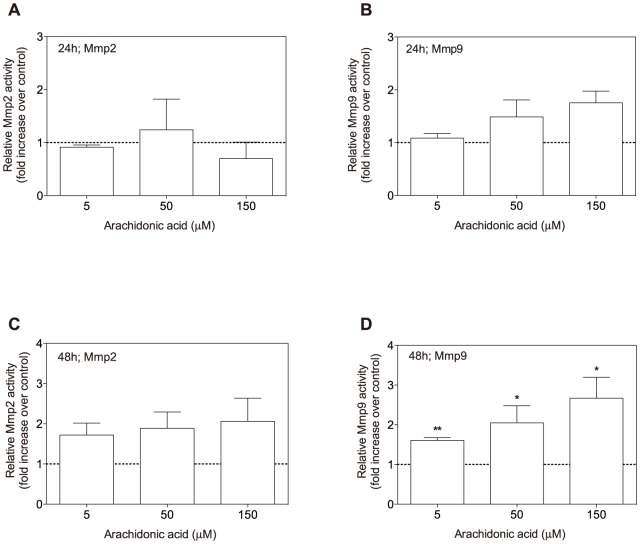
In-vitro exposure of scales to arachidonic acid. Semiquantitative analysis of gelatinolytic matrix metalloproteinases of culture medium after *in vitro* exposure of ARA for 24 hours (***A*** and ***B***) and 48 hours (***C*** and ***D***). ***A***: ARA has no effect on Mmp2 activity after 24 hours of culture. ***B***: A transient, non-significant increasing trend is measured in Mmp9 activity with increasing ARA concentration. ***C***: Stimulatory effects of ARA on Mmp2 are not significant. ***D***: ARA significantly increases MMP9 after 48 hours of culture, with a concentration-dependent trend. Activities of Mmp2 (***A***, ***C***) and Mmp9 (***B***, ***D***) are presented relative to the Mmp activity of samples taken from control cultures. Samples are expressed as mean ± SEM of 2 (24 h) or 6 replicates (48 h). Statistical analysis was done with the Student's *t*-test or Mann-Whitney U-test when results were not normally distributed (* p<0,05, ** p<0,01).

To assess whether ARA can affect scale cells directly, or needs to be metabolized elsewhere in the body, we exposed scales to ARA *in vitro*. After 24 hours *in vitro* ARA exposure (5, 50 and 150 µM) of scales in culture, no effects were observed on Mmp2 (figure 6A) and Mmp9 activities (figure 6B). However, after 48 hours both Mmp2 (not significant, P = 0.123) and Mmp9 activities had increased in the presence of arachidonic acid (figure 6C, D).

## Discussion

Elasmoid scales of (zebra-)fish are an ideal model system to study how nutrients subtly modulate skeletal physiology *in vitro* as well as *in vivo*. The use of ontogenetic and regenerating scales provides us with different resolutions as the rate of matrix formation and resorption is different between ontogenetic and regenerating scales [36], [38]. In the present study we show that six weeks of feeding zebrafish with either low or high levels of ARA in the diets dramatically changes whole body fatty acid profiles according to the respective diet. As a consequence, mineralization of scales was negatively affected and osteoclast activity and markers of bone turnover were increased in scales of fish fed the high ARA diet.

Despite large differences in whole body ARA levels between the two groups, only subtle (yet significant) differences were observed in terms of mineral composition. As discussed in previous work, the small size of the zebrafish scale poses a technical limitation to measure parameters of bone mineral density (BMD); best alternatives are histomorphometry of demineralization and calculation of the Ca:P molar ratio [36], [44]. The Ca:P ratio was decreased in ontogenetic scales of fish fed the high ARA diet, which we take as evidence that (scale) mineralization is affected by dietary ARA. Lower Ca:P ratios of biological samples (<1.6, the ratio for pure hydroxyapatite) generally point towards altered crystalline phase, impurities or defects, all of which make the mineral less stable and more easy to dissolve by osteoclasts [45]. Scales grow continuously throughout life and formation and resorption is ongoing, albeit at a very low pace [38]. The effect seen is likely the result of delicate changes in scale growth and demineralization and could be mediated via increased incorporation of *e.g.* magnesium (leading to whitlockite) and/or carbonate (defective apatites), or via increased deposition of amorphous calcium phosphates [44]. During scale regeneration, the Ca:P ratio increased from approximately 1.2 early in regeneration (day 4), to a value of approximately 1.4 when mineralization of the scale matrix progressed and the scale ‘matured’. This increase in Ca:P ratio is typical for the early phase of scale regeneration and is consistent with previous findings in zebrafish [36], [38] and sea bass (*Dicentrarchus labrax*) [46]. We further observed a higher Ca:P ratio in 7-days-old regenerating scales of fish fed the high ARA compared to those fed the low ARA diet. As the increase in Ca:P ratio is typical for elasmoid scale maturation, the increase measured in the high ARA group may suggest that ARA increases the rate of development and maturation.

For ontogenetic scales, increased osteoclastic activity in the high ARA group is particularly evident from the histomorphometric analyses. An increase in the area positive for TRAcP activity was detected, as well as increased resorption (in total perimeter and absolute number of pits). This indicates that osteoclasts degrade more and/or longer stretches of scale matrix. Bone resorption, the catabolic phase of the bone remodeling process, is characterized by increased proteolytic activity to degrade matrix following mineral dissolution [47], [48]. Although no significant differences were found in expression of genes encoding proteins involved in bone resorption (and formation), actual secretion of Mmps enzymes was significantly increased in the high ARA group. This shows that the observed effects on the mineralized matrix are the result of increased osteoclast activity and do not depend on increased expression of osteoclast genes. Moreover, a small increase in osteoclastic activity or number may already account for the substantial increase of resorption.

Increased matrix resorption is a natural aspect of scale regeneration [38] and likely obscures the subtle differences that may be the result of the different diets. We did however find evidence of increased osteoclastic activity on the level of gene expression; *rankl* and *mmp9* were upregulated in 4-day-old regenerating scales of fish fed the high ARA diet. At day 4 of regeneration the increased enzymatic activity in regenerating scales of fish fed the high ARA was also increased near significance. For Mmp9, the increased enzymatic activity was maintained until day 7 and is apparently sustained (for a certain period) when gene expression already declines. The effects of high dietary ARA levels on both *mmp* genes and *rankl* (and subsequent osteoclastic activities) in regenerating scales are in line with the results found in several mammalian *in vitro* studies [17], [22], [23].

In the present study, both enzymatic activity of Mmps and gene expression showed a high degree of individual variation, which is likely intrinsic to differences in size and cellular activity between individual scales. Zebrafish possess hundreds of elasmoid scales, which allows for substantial individual variation in the scale compartment that apparently functions as a whole [38]. In this study, the variation in enzymatic activities appears to partly conceal the effects of ARA and the resolution of band-intensity measurements is fairly low. Yet in all scales analyzed, it is apparent that more proteolytic enzyme was secreted from the osteoclasts associated with scales from fish fed the high ARA diet.

The effects of ARA on proteolytic activity were much more pronounced *in vitro*, when scales were exposed directly to ARA (5–150 µM). Already at the lowest concentration applied (5 µM), enhanced secretion of Mmp9 was seen, in line with *in vitro* studies on mammalian osteoclast-like cells; Yuan and coworkers showed that 10 µM ARA enhanced osteoclastogenesis directly in bone marrow-derived macrophages through the ARA metabolite prostaglandin E2 (PGE2) [51]. Using scales from goldfish (*Carassius auratus*), Omori and colleagues showed that PGE2 increased both osteoclastic and osteoblastic activity *in vitro* as well as *in vivo* [26]. Taken together, the observations made on ontogenetic scales show that dietary ARA has a mild but significantly stimulatory effect on osteoclastic matrix degradation in scales. From the *in vivo* and *in vitro* experiments combined, we conclude that ARA exerts direct effects on scale cells, most likely orchestrated by the cyclooxygenase product PGE2 [52].

Several studies show effects of ARA on differentiation and activity of mammalian osteoblasts, suggesting that ARA has a negative effect on matrix deposition and mineralization [9], [22]. Recently this was also demonstrated for osteoblasts of teleost fish; treatment of seabream (*Sparus auratus*) osteoblasts with 100 µM ARA (and EPA) increased proliferation but decreased mineralization *in vitro* [24]. In contrast, one of the few effects we observed on osteoblast-associated genes is the increased expression of *sp7* in 4-days-old regenerating scales. In addition, we observed increased expression of the *rankl* gene, which encodes a powerful osteoblast-derived stimulator of osteoclast activity and according matrix resorption [49], [50]. Scale regeneration is characterized by a typical, phased gene expression profile that includes increased expression of *sp7*, *rankl* and *mmp9* [36], [38]. Expression of these genes is increased on day 4 of scale regeneration in the high ARA group, while scales of the low ARA group reached similar expression levels on day 7, when expression had significantly increased compared to day 4. We take this as evidence that the typical and transient phased expression profile is accelerated in the high ARA group. Accordingly, the effects on mineral composition of the matrix at day 7 are the result of the combined (and delayed) mineralizing and resorptive activity up to that moment.

Considering the large differences in ARA content between the two feeds (and as a consequence in the fish), the effects on mineral deposition are relatively small, indicative of the subtle yet important and robust modulatory role of ARA/PGE2 in bone physiology. Data originating from *in vitro* experiments are usually more profound as they focus either on osteoblasts or osteoclasts separately. It is however not uncommon for these effects to be more subtle in intact bone tissue and in *in vivo* experiments, as the osteoblasts and osteoclasts are known to maintain tightly controlled balance resulting from bidirectional communication [12], [14], [53]. Here, the *in vitro* scale model has an advantage over cell monocultures, as the scale culture benefits from the presence of both osteoblasts and osteoclasts, a natural co-culture [29]. Our results are in agreement with the in-vitro work of Omori and colleagues, who showed that PGE2 (an active metabolite of ARA) increases enzymatic activity of the osteoblast-marker alkaline phosphatase in goldfish elasmoid scales [26]. Although their study shows that PGE2 is able to activate scleroblasts (referred to as osteoblasts), they also provide strong evidence that PGE2 stimulates osteoclast activity. Similarly, from our study it follows that ARA affects both cell types, which may explain the different outcomes in existing bone and new-forming bone, with the characteristic high level of expression of osteoblast-related genes in the latter [36].

Another consideration is a hyperbolic relation between ARA concentration and effects exerted. Interestingly, Senegalese sole (*Solea senegalensis*) larvae fed *Artemia* enriched with different levels of ARA showed the highest increase in growth and mineralization in the group receiving an intermediate concentration of ARA (5.7% ARA of total fatty acid, TFA) compared to groups receiving a lower (2% of TFA) and higher (7.3% of TFA) concentration [1]. This effect was confirmed in sea bream and sea bass [54], [55], indicating a possible critical level of ARA needed to sustain optimal growth. Thus it could be speculated that the effects seen on scale development *in vivo* in the present study is a result of attenuated growth in the low ARA group and not necessarily the direct effect of free ARA on the skeleton, as demonstrated *in vitro* in previous as well as in the current study. To the best of our knowledge there are no reports on optimal fatty acid composition for zebrafish feed to which we can relate the fatty acid composition of our experimental diets. Future studies will address hyperbolic relationships between ARA treatments and their effects on bone physiology. It is clear though that ARA has a modulatory effect on scale metabolism predominantly mediated via enhanced osteoclastic matrix degradation. Hence, ARA requirements in different species and of developmental stages need attention to secure fish welfare in aquaculture.

## Conclusion

We conclude that an ARA-enriched diet, and consequent changes in whole-body fatty acid profile, influences scale mineralization and remodeling in existing (ontogenetic) and regenerating scales. The effects found in this study correlate with the skeletal deformities found as a result of sub-optimal dietary fatty acid composition. We thus conclude that the scale model can be used in aquaculture to quickly predict the effects of dietary composition on the entire skeleton. Feeding trials are notoriously difficult as they depend strongly on dose and composition of fatty acid supplementation (and many more variables). Many such studies though point towards increased turnover of bone as results of dietary fatty acid supplementation [21]. Small differences in mineralization of skeletal tissues are known to lead to malformations and fractures, especially when subjected to great forces [5], [7], [56]. We show that studies on elasmoid scales of zebrafish, including the regeneration paradigm, may provide an excellent tool for future studies to elucidate underlying molecular mechanisms of (dietary) fatty acids on subtle aspects of skeletal biology.

## Supporting Information

File S1 **Supporting file including Tables S1–S4.** **Table S1:** Composition of mineral mix. **Table S2:** Composition of vitamin mix. **Table S3:** Composition of amino acid mix. **Table S4:** Primer sequences of target genes used in quantitative PCR.
(DOCX)
