# Supplementary material for: Arachidonic Acid Enhances Turnover of the Dermal Skeleton: Studies on Zebrafish Scales
Source: PLoS One. 2014 Feb 19;9(2):e89347. doi: 10.1371/journal.pone.0089347 (PMC3929718; doi:10.1371/journal.pone.0089347)
Supplement: File S1 — Supporting file including Tables S1–S4. Table S1: Composition of mineral mix. Table S2: Composition of vitamin mix. Table S3: Composition of amino acid mix. Table S4: Primer sequences of target genes used in quantitative PCR. (DOCX) [file pone.0089347.s001.docx]

**Table S1: composition of mineral mix**

| **Ingredients** | **Content (g/kg)** |
| --- | --- |
| CaHPO_4_.2H_2_O | 15 |
| CoCl2.6H2O | 0.002 |
| CuSO4.5H2O | 0.01 |
| FeSO4.7H2O | 0.4 |
| KCl | 15 |
| KI | 0.01 |
| MgSO4.7H2O | 5 |
| MnSO4.H2O | 0.08 |
| NaCl | 4.148 |
| ZnSO4.7H2O | 0.2 |
| Yeast (Zn source) | 0.15 |

**Table S2: composition of vitamin mix**

| **Ingredients** | **Content (mg/kg)** |
| --- | --- |
| Vitamin A | 10 |
| Vitamin D3 | 4 |
| Vitamin E (50%) | 130 |
| Vitamin K (50%) | 10 |
| Vitamin C (35%) | 350 |
| Choline | 4000 |
| Thiamin mononitrate | 15 |
| Riboflavin (80%) | 19 |
| Pyridoxin.HCl | 20 |
| Niacin | 200 |
| Inositol | 400 |
| Folic acid | 5 |
| Ca-pantothenate | 60 |
| Biotin | 50 |
| B12 (0.1%) | 30 |
| Casein | 4697 |

**Table S3: composition of amino acid mix**

| **Ingredients** | **Content (mg/kg)** |
| --- | --- |
| Betaine | 0.5 |
| Glycine | 3 |
| Alanine | 0.8 |
| Arginine | 5 |
| Methionine | 5 |
| Valine | 3 |
| Taurine | 0.2 |
| Glutamic acid | 0.3 |
| Aspartic acid | 0.3 |
| Serine | 0.3 |
| Lysine | 8.1 |
| Histidine | 3 |
| Tryptophan | 4.5 |
| Isoleucine | 5 |
| Leucine | 5 |
| Phenylalanine | 4 |
| Threonine | 2 |

**Table S4: Primer sequences of target genes used in quantitative PCR.**

| **Gene** | **Accession number** | **Sequence (‘5-’3)** |
| --- | --- | --- |
| *tuba1l* | NM_001105126.2 | FW: GGTGCCCTCAATGTGGATCT |
|  |  | RV: GCCACAGAGAGCTGCTCATG |
| *sp7 (osterix)* | AY380818.1 | FW: CCCATTCTCCACTTTCCAGA |
|  |  | RV: TGGATGCCTTTGTACCATGA |
| *rpl13a* | NM_212784 | FW: TCTGGAGGACTGTAAGAGGTATGC |
|  |  | RV: AGACGCACAATCTTGAGAGCAG |
| *cathepsin K* | NM_001017778 | FW: ATGATCTGGGCATGAACCAT |
|  |  | RV: CCGAAGTGACGTATCCCAGT |
| *osteocalcin* | NM_001083857 | FW: TGACGTGGCCTCTATCATCA |
|  |  | RV: TTTATAGGCGGCGATGATTC |
| *matrix metalloproteinase 9 (mmp9)* | NM_213123.1 | FW: TCATGATCTCTGCGAAGTGG |
|  |  | RV: CCGAGCTTCTCGATTTTACG |
| *Rankl (tnfsf11)* | ZDB-GENE-090805-2 | FW: TAGTGTGGCGATTCTGTTGC |
|  |  | RV: ATTGGAAGGTGAGCTGATGG |
